# The grapevine guard cell-related VvMYB60 transcription factor is involved in the regulation of stomatal activity and is differentially expressed in response to ABA and osmotic stress

**DOI:** 10.1186/1471-2229-11-142

**Published:** 2011-10-21

**Authors:** Massimo Galbiati, José Tomás Matus, Priscilla Francia, Fabio Rusconi, Paola Cañón, Consuelo Medina, Lucio Conti, Eleonora Cominelli, Chiara Tonelli, Patricio Arce-Johnson

**Affiliations:** 1 Dipartimento di Scienze Biomolecolari e Biotecnologie, Università degli Studi di Milano, Via Celoria 26, 20133 Milano, Italy; 2 Fondazione Filarete, Viale Ortles 22/4, 20139, Milano, Italy; 3 Pontificia Universidad Católica de Chile, Departamento de Genética Molecular y Microbiología. Alameda 340. Santiago, Chile; 4 Centre for Research in Agricultural Genomics (CRAG), 08193 Barcelona, Spain; 5 Istituto di Biologia e Biotecnologia Agraria, CNR; Milano, Italy

## Abstract

**Background:**

Under drought, plants accumulate the signaling hormone abscisic acid (ABA), which induces the rapid closure of stomatal pores to prevent water loss. This event is trigged by a series of signals produced inside guard cells which finally reduce their turgor. Many of these events are tightly regulated at the transcriptional level, including the control exerted by MYB proteins. In a previous study, while identifying the grapevine R2R3 MYB family, two closely related genes, *VvMYB30 *and *VvMYB60 *were found with high similarity to *AtMYB60*, an Arabidopsis guard cell-related drought responsive gene.

**Results:**

Promoter-GUS transcriptional fusion assays showed that expression of *VvMYB60 *was restricted to stomatal guard cells and was attenuated in response to ABA. Unlike *VvMYB30*, *VvMYB60 *was able to complement the loss-of-function *atmyb60-1 *mutant, indicating that *VvMYB60 *is the only true ortholog of *AtMYB60 *in the grape genome. In addition, *VvMYB60 *was differentially regulated during development of grape organs and in response to ABA and drought-related stress conditions.

**Conclusions:**

These results show that VvMYB60 modulates physiological responses in guard cells, leading to the possibility of engineering stomatal conductance in grapevine, reducing water loss and helping this species to tolerate drought under extreme climatic conditions.

## Background

Grapevine (*Vitis vinifera *L.) is a fruit crop traditionally subjected to moderate or severe water stress, as this is an efficient strategy to improve fruit and wine quality (reviewed in [1,2]). Vitis species adapt well to drought conditions due to good osmotic adjustment, large and deep root systems, efficient control of stomatal aperture and xylem embolism [3,4]. The strength and timing of these responses varies between different cultivars and major differences in water stress tolerance can be found when compared to other species or hybrids from the Vitis genus [5]. Although these genotype-related variations involve different aspects of the physiology of the plant, they are largely linked to differences in stomatal conductance (*g*_s_) [6]. Stomata are microscopic pores distributed on the surface of leaves and stems, surrounded by two highly specialized guard cells. The opening and closure of the pore, in response to internal signals and environmental cues, allows the plant to cope with the conflicting needs of ensuring adequate uptake of CO_2 _for photosynthesis and preventing water loss by transpiration [7]. Under drought, abscisic acid (ABA) is accumulated, inducing rapid stomatal closure to limit water loss.

Increasing evidence indicates a role for transcription factors belonging to the R2R3 MYB subfamily as key modulators of physiological responses in stomata [8,9]. In particular, *AtMYB60 *has been shown to be differentially expressed in guard cells in response to ABA, and the loss-of function *atmyb60-1 *mutant displays constitutive reduction of light-induced stomatal opening and enhanced tolerance to dehydration [10]. Guard cell-specific MYB genes are thus focal points in understanding stomatal regulation in plants and represent molecular targets to modulate guard cell activity to improve crop survival and productivity during drought.

The grapevine genome has been estimated to contain a total of 279 MYB genes [11], of which 108 belong to the R2R3 subfamily [12]. A phylogenetic tree, constructed with the complete grape, Arabidopsis and rice R2R3MYB subfamilies, showed that many genes sharing similar functions were clustered in the same phylogenetic groups. Some of these clades were conserved in gene copy number (e.g. those related to trichome development) while in those controlling flavonoid synthesis several expansions events may have occurred [12].

In this work, we report the identification of two close homologues of the guard cell-related *AtMYB60 *gene in the grape genome, namely *VvMYB60 *and *VvMYB30*. Analysis of gene expression in grape tissues revealed that both *VvMYB60 *and *VvMYB30 *were expressed in green tissues and developing seeds. As opposite to *VvMYB30*, *VvMYB60 *transcript abundance was greatly reduced by ABA and osmotic stress. A GUS reporter gene approach in Arabidopsis showed that activity of the *VvMYB60 *promoter was restricted to stomatal guard cells and was down-regulated by ABA. Comparative analysis of regulatory regions revealed the presence of common guard cell-specific motifs in the promoters of the grape and Arabidopsis *MYB60 *genes. Finally, *VvMYB60*, unlike *VvMYB30*, fully complemented the stomatal defects of the *atmyb60-1 *mutant, thus indicating that *VvMYB60 *is a functional ortholog of the Arabidopsis *AtMYB60 *stomatal regulator.

## Results

### Phylogenetic relationships of MYB60 homologues

As a first approach to identify grape homologues of the AtMYB60 transcription factor, we searched the 108 R2R3 MYB proteins identified in the *Vitis vinifera *PN40024 genome [12], for the presence of a distinctive C-terminal motif (CtM2, YaSS^T^/_A_eNI^A^/_S_^R^/_K_Ll), found in members of Subgroup 1 of the Arabidopsis MYB family [13]. This subgroup includes: AtMYB60, regulating light-induced stomatal aperture [10,14]; AtMYB30, related to the regulation of brassinosteroid-induced gene expression [15] and to the biosynthesis of very-long-chain fatty acids involved in hypersensitive cell death [16]; AtMYB96, an ABA/auxin cross-talker, mediating ABA signaling during drought stress and involved in promoting pathogen resistance [17,18] and AtMYB94, whose function is still unknown.

**Figure 1:**
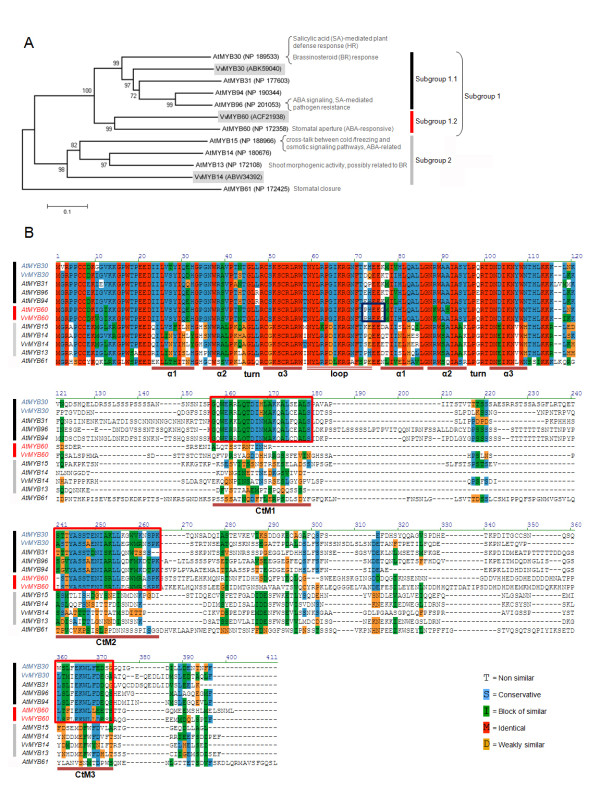
**Analysis of grape and Arabidopsis MYB homologues within Subgroup 1**. (**A**), Phylogenetic relationships between Arabidopsis and grape subgroups 1 and 2 of R2R3 MYB factors, as described by Kranz *et al*., [13]. A consensus rooted tree was inferred using the Maximum Parsimony method, constructed with MEGA4^® ^software. (**B**) Alignment of deduced amino acid sequences of subgroup 1 and 2 R2R3 MYB homologues from Arabidopsis and grape. The R2 and R3 repeats lie between the three alpha helices of each repeat. Boxes represent the C-terminal motifs CtM1, CtM2 and CtM3 (red boxes) conserved in members of subgroup 1 and the PHEEG signature (blue box), distinctive of AtMYB60 and VvMYB60 (subgroup 1.2). Amino acid residues are shaded in different colors, as indicated in the legend. Dots represent gaps introduced to improve the alignment.

Our search yielded two grape close homologues in the grape genome version 12x: the annotated gene models GSVIVT01008005001 (protein accession ABK59040) and GSVIVT01029904001 (protein accession ACF21938). A parsimony consensus tree was constructed to investigate the phylogenetic relationships within these grape proteins and members of Arabidopsis R2R3 MYB Subgroup 1. Subgroup 2 was also included as some of its members are involved in drought responses and ABA signaling [19,20]. From this subgroup, a grape *MYB14 *homologue had also been previously isolated [12]. AtMYB61, regulating stomatal activity [21], but not belonging to any of these subgroups, was included as an out-group. As shown in Figure 1A, the two grape proteins ACF21938 and ABK59040 clustered with members of the Arabidopsis Subgroup 1. Interestingly, AtMYB60, the most distant member of Subgroup 1, was more closely related to the grape protein accession ACF21938 than to the other members of the subgroup (AtMYB30, 31, 94 and 96). On the other hand, the grape accession protein ABK59040 was closely related to AtMYB30 and AtMYB31 and to a lesser extent to AtMYB94 and AtMYB96 (Figure 1A). Hereafter, we will refer to ACF21938 and ABK59040 as VvMYB60 and VvMYB30, respectively. Based on these results, we further divided Subgroup1 into Subgroup 1.1, (AtMYB30, AtMYB31, AtMYB94 and AtMYB96) and Subgroup 1.2 (AtMYB60 and VvMYB60) (Figure 1A).

As expected, all the proteins included in the tree disclosed a highly conserved R2R3 DNA binding domain (Figure 1B). The identity between the R2R3 domain of AtMYB60 and VvMYB60 and VvMYB30 was 99%, and 90%, respectively. In addition, AtMYB60 and VvMYB60 disclosed a distinctive PHEEG signature, encompassing the two highly conserved glutamic acid residues, located in the loop connecting the R2 and R3 repeats (Figure 1B). The complete protein sequence of AtMYB60 showed 51% amino acid identity to VvMYB60, and 48% identity to VvMYB30. All of these proteins share two C-terminal motifs (CtM2 and CtM3) which are only found in subgroup 1. In addition, AtMYB30, 31, 96 and 94 possess a third MYB domain (CtM1), which is absent in AtMYB60 and VvMYB60 (Figure 1B). The function of these C-terminal domains is still unknown although they might reflect the functional differences between subgroups 1.1 and 1.2. We determined the precise gene structure of both *VvMYB30 *and *VvMYB60*, by comparing the complete coding sequence with the full length cDNA sequence, amplified from Pinot noir PN40024 genomic DNA and leaf cDNA, respectively (Additional file 1). It was interesting to note that in the 12x version of the grape genome, GSVIVP01008005001, representing the *VvMYB60 *gene model, was misannotated in terms of exon number. Indeed, our results indicate the presence of three exons, as opposed to the five exons predicted by the gene model, thus revealing a conserved exon/intron organization for *VvMYB30*, *VvMYB60 *and *AtMYB60 *(Additional file 1). Based on gene structure, MYB genes have been classified in four different groups [12]. *VvMYB30*, *VvMYB60 *and *AtMYB60 *all belong to Group I, which contains genes with a characteristic R2 domain split between exons 1 and 2, and a R3 domain split between exons 2 and 3 (Additional file 1). The biggest differences in lengths were found in the first intron and the third exon, which were longer in the grape genes compared to *AtMYB60*.

### Expression of *VvMYB30 *and *VvMYB60 *in grape tissues and in response to hormonal and stress factors

**Figure 2:**
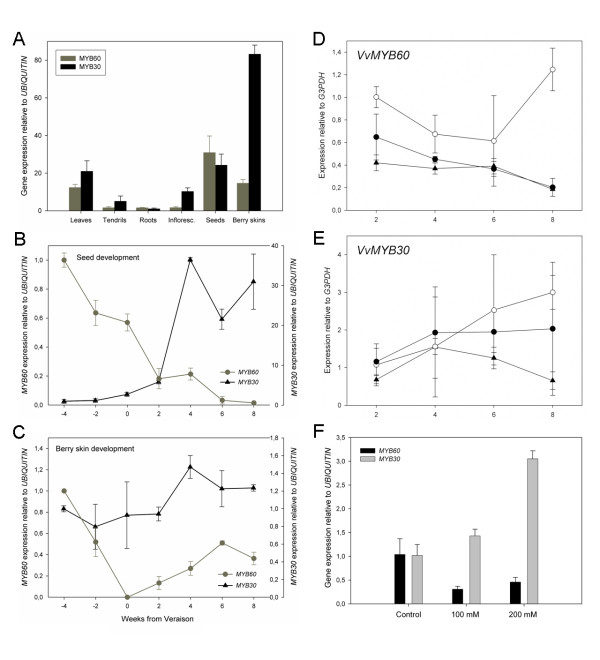
**Gene expression profiles of *VvMYB60 *and *VvMYB30 *in different plant tissues and in response to ABA**. (**A**) Expression in grapevine organs. (**B**) and (**C**) Expression throughout berry seed and skin development (X-axis corresponds to weeks from veraison). Each gene was independently normalized. (**D**) and (**E**) Expression in response to applied ABA in leaf disks. X-axis corresponds to hours after ABA application. White circle: Mock solution, black circle: 50 μM ABA, black triangle: 100 μM ABA. (**F**) Expression changes of *VvMYB60 *and *VvMYB30 *in grapevine plantlets subjected to salt stress conditions. Each gene was independently normalized against its control treatment (standard MS, with 3 mM NaCl). Means and SD are the result of three independent replicates. Reference genes (*UBIQUITIN *and *GLYCERALDEHYDE 3-PHOSPHATE DEHYDROGENASE) *were differently selected according to the experimental condition in which they presented less variation among samples and assuming they behaved similarly as described in [43].

qPCR analysis of gene expression in different grape organs indicated that *VvMYB30 *and *VvMYB60 *transcripts were most abundant in leaves, seeds and ripened berry skins (Figure 2A). Interestingly, *VvMYB30 *and *VvMYB60 *revealed completely opposite expression patterns during seed development; while *VvMYB60 *expression was gradually down-regulated towards the onset of ripening (*veraison*), *VvMYB30 *expression was rapidly activated (Figure 2B). During berry skin development, *VvMYB60 *expression also showed a dramatic decrease to full repression at *veraison*, followed by a slight increase towards ripening (Figure 2C). In this tissue, *VvMYB30 *was mostly constantly expressed throughout the green and ripening stages (Figure 2C).

In Arabidopsis, it has been shown that the expression of the *AtMYB60 *gene is rapidly down-regulated following treatment with ABA [10]. We thus analysed the expression of the grape genes in leaves treated with 50 or 100 μM ABA (Figure 2D and 2E). As reported in Figure 2D, V*vMYB60 *showed a significant decrease in expression levels in both 50 and 100 μM ABA treated samples, when compared to the mock treated leaves. Conversely, *VvMYB30 *did not show any change in expression after exposure to the hormone under these conditions (Figure 2E). To further investigate the expression of *VvMYB60 *and *VvMYB30 *in response to osmotic stresses, we designed an *in vitro *long-term salt stress experiment. Nodal explants were placed vertically on sterile MS media supplemented with 3 (standard), 100 or 200 mM NaCl. Explants were maintained for a month in a growth chamber until roots and/or leaves were visible and fully expanded. At the end of the experiment, plantlets from the 100 mM NaCl treatment had a small radicule and high leaf anthocyanin accumulation, as a clear sign of stress in the plant, while plants at 200 mM showed more severe symptoms, including systemic wilting and brown pigmentation (Additional file 2). Under these conditions, *VvMYB60 *and *VvMYB30 *showed opposite responses to the increasing salt concentrations; while *VvMYB60 *expression was reduced five-fold at both concentrations when compared to the control treatment, *VvMYB30 *expression increased three-fold on addition of 200 mM NaCl (Figure 2F).

### Activity of the *VvMYB30 *and *VvMYB60 *promoters in Arabidopsis transgenic lines

We employed a reporter gene approach in the heterologous model system *Arabidopsis thaliana *to investigate the activity of both *VvMYB30 *and *VvMYB60 *promoters. A region of approximately 2 kb located upstream of the ATG codon of *VvMYB30 *and *VvMYB60 *was fused to the β*-glucuronidase *(*GUS*) reporter gene and the resulting *pVvMYB30:GUS *and *pVvMYB60:GUS *constructs were introduced in Arabidopsis by *Agrobacterium*-mediated transformation [22].

**Figure 3:**
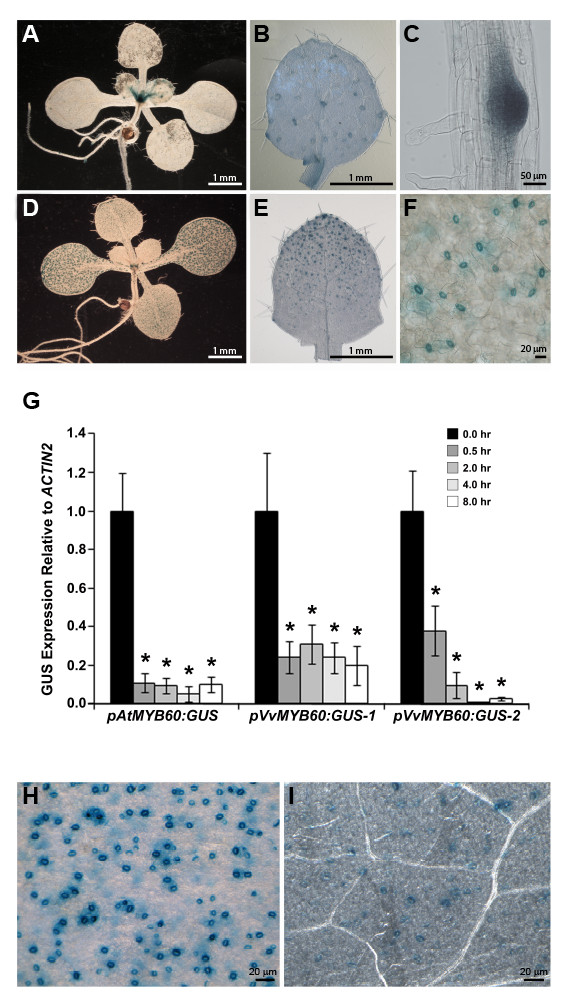
**Activity of the grape *VvMYB60 *promoter is localized to guard cells in Arabidopsis, and is down-regulated by ABA**. (**A**) 15-day-old *pVvMYB30:GUS *seedling. (**B**) Magnification of leaf primordia in (**A**), showing staining at the base of trichomes. (**C**) Detail of an emerging later root. (**D**) 15-day-old *pVvMYB60:GUS *seedling. (**E**) Magnification of leaf primordia in (**D**), showing staining of differentiating stomata. (**F**) Detail of a *pVvMYB60:GUS *mature leaf, showing staining of fully differentiated stomata. (**G**) qPCR analysis of GUS expression in response to 100 μM ABA, in two independent *pVvMYB60:GUS *lines (mean ± SD, n = 3). A transgenic line carrying the 1.3 kb Arabidopsis *MYB60 *promoter fused to GUS (*pAtMYB60:GUS*) was used as a control. Total RNA samples were extracted at the time points indicated (hours). Relative GUS transcript levels were determined using gene-specific primers and normalized to the expression of the *AtACTIN2 *gene (At3g18780). Asterisks indicate values significantly different from the untreated control (*P < 0.001*, *t-test*). (**H**) and (**I**) Histochemical analysis of GUS expression in *pVvMYB60:GUS *leaves in response to ABA. (**H**) GUS staining of stomata in a control leaf. (**I**), GUS staining of stomata following 6 hours of exposure to 100 μM ABA.

We assessed the cell and tissue specificity of reporter gene expression in ten independent T3 transgenic lines for each *promoter:GUS *combination. Fifteen-day-old *pVvMYB30:GUS *seedlings displayed expression of the reporter at the shoot apex, at the base of trichomes located on leaf primordia, and in the emerging lateral roots (Figure 3A, B and 3C). At the same developmental stage, *pVvMYB60:GUS *seedlings showed GUS expression exclusively in guard cells distributed on cotyledons, hypocotyls and developing leaves (Figure 3D and 3E). No expression of the reporter gene was detected in rosette leaves from mature *pVvMYB30:GUS *plants, even after prolonged incubation of plant tissues in the GUS solution (data not shown). On the other hand, we observed guard cell-specific signals in mature leaves of *pVvMYB60:GUS *plants, consistent with the GUS profile observed in young seedlings (Figure 3F).

Next, we investigated expression of the reporter in flowers and siliques from adult plants. Prior to pollination, *pVvMYB30:GUS *flowers revealed a diffuse staining of carpels and stigmatic tissues (Additional file 3A). We did not observe GUS expression in pre- and post-fertilization flowers from most *pVvMYB60:GUS *lines. In two transgenic lines, a weak staining was occasionally detected in stamens, at the interface of filaments and anthers (Additional file 3B). Finally, we did not detect expression of the reporter in developing seeds from either *pVvMYB30:GUS *or *pVvMYB60:GUS *transgenic lines (Additional file 3C).

Expression of both the endogenous Arabidopsis and grape *MYB60 *genes is rapidly down-regulated following treatment with ABA [10] (Figure 2D). We thus investigated changes in GUS expression in the *pVvMYB60:GUS *lines in response to exogenous applications of this hormone, using both qPCR and histochemical analyses. A previously described transgenic line carrying a transcriptional fusion between the Arabidopsis *AtMYB60 *promoter and the reporter GUS (*pAtMYB60:GUS*) was used as a control for the experiment [10]. As expected, qPCR analysis of GUS expression revealed a significant and rapid decrease in the accumulation of GUS transcripts in the control *pAtMYB60:GUS *plants following exposure to ABA (*P < 0.001*) (Figure 3G). We observed a comparable reduction in GUS expression in two independent *pVvMYB60:GUS *lines, that were randomly selected for the qPCR experiment (*P < 0.001*) (Figure 3G). Staining of rosette leaves excised from all the ten *pVvMYB60:GUS *lines, before and after treatment with ABA, confirmed the negative effect of the hormone on the activity of the *VvMYB60 *promoter (Figure 3H and 3I). Conversely, treatment of *pVvMYB30:GUS *plants with ABA did not significantly affect the expression of the reporter (data not shown).

### Occurrence of guard cell-specific motifs in the *VvMYB60 *promoter

The conserved activity of the Arabidopsis and grape *MYB60 *promoters, as emphasized by the analysis of the corresponding *promoter:GUS *transgenic lines, suggests that these two regulatory regions might share common *cis*-elements responsible for the guard cell-specific expression of the reporter. Previous evidence indicates a role for DNA consensus sequences for DOF-type transcription factors ([A/T]AAAG) as guard cell-specific *cis*-active enhancers [23]. Specifically, clusters of at least three [A/T]AAAG motifs located on the same strand within a region of at most 100 bp were identified as putative guard cell-specific *cis*-regulatory elements [24].

The Arabidopsis *AtMYB60 *promoter contains multiple [A/T]AAAG clusters, of which the most proximal to the translation start codon (-143 bp), is necessary and sufficient to drive expression in guard cells (Cominelli, unpublished results) (Additional file 4). We thus searched the grape *VvMYB30 *and *VvMYB60 *promoters for the occurrence of [A/T]AAAG oligonucleotides, in a region of 300 bp upstream of the translation start site. We identified a cluster of three [A/T]AAAG motifs in the *VvMYB60 *promoter, located at -169 bp from the ATG codon of the endogenous gene, a distance comparable to the position of the guard cell regulatory element found in the promoter of *AtMYB60*. Consistent with the lack of activity in Arabidopsis guard cells no [A/T]AAAG clusters were identified in the promoter of the grape *VvMYB30 *gene (Additional file 4).

The cellular specificity of gene expression has been investigated for a very limited number of grape genes. Among these, *VvSIRK*, encoding a K^+ ^channel, has been reported to be specifically expressed in guard cells [25]. Interestingly, we discovered an [A/T]AAAG cluster upstream of the translation start codon (-200 bp) of *VvSIRK*, in the opposite orientation relative to the direction of transcription (Additional file 4).

### Functional complementation of the Arabidopsis *atmyb60-1 *mutant by *VvMYB60*

A null allele of the Arabidopsis *AtMYB60 *gene (*atmyb60-1*) displays constitutive reduction of the opening of the stomatal pores and reduced water loss during drought [10]. Interestingly, despite its increased tolerance to dehydration relative to the wild type, the *atmyb60-1 *mutant does not show obvious alterations in the sensitivity of guard cells to ABA [10].

We used the *atmyb60-1 *allele to investigate the role of *VvMYB60 *in the regulation of stomatal activity and to explore the conservation of the *MYB60 *gene function between grape and Arabidopsis. To this end, we introduced the full length *VvMYB60 *cDNA in transgenic mutant plants (*atmyb60-C60 *lines) to assess the ability of the grape gene to rescue the stomatal defects of the *atmyb60-1 *allele. As a control for the complementation, we generated a second series of transgenic plants, in which we transformed the full length *VvMYB30 *cDNA in the *atmyb60-1 *background (*atmyb60-C30 *lines). It is important to note that the two *VvMYB30 *and *VvMYB60 *promoters displayed very different patterns of activity in Arabidopsis (Figure 3A, B, C, D, E and 3F). Hence, for a more robust and reliable comparison of the two grape genes in the *atmyb60-1 *background we used the 1.3 kb *AtMYB60 *promoter [10] to drive the expression of *VvMYB30 *and *VvMYB60 *in guard cells. Three independent transgenic mutant lines with a single insertion locus and comparable levels of expression of the transgene were selected for further analysis of each grape gene (Additional file 5).

**Figure 4:**
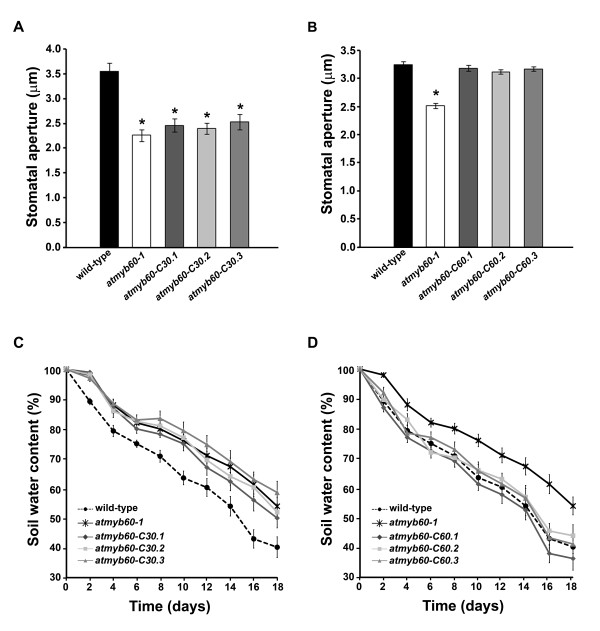
**The grape *VvMYB60 *gene complements stomatal defects of the Arabidopsis *atmyb60-1 *mutant**. (**A**) and (**B**) Stomatal aperture assay in wild-type, *atmyb60-1 *and three independent transgenic mutant lines carrying the *VvMYB30 *(**A**) or the *VvMYB60 *(**B**) gene, under the control of the guard cell-specific *AtMYB60 *promoter. Measurements were performed on epidermal strips excised from dark-adapted plants and exposed to light for 4 hr. Each bar indicates mean ± SD of three separate experiments (n = 100 stomata per bar). The asterisk indicates values significantly different from wild-type (*P < 0.001*, *t-test*). (**C**) and (**D**) Changes in soil water content during drought stress treatment of wild-type, *atmyb60-1 *and three independent mutant lines complemented with the *VvMYB30 *gene (**C**), or the *VvMYB60 *gene (**D**). Plants grown under normal watering conditions for 20 days were drought stressed by complete termination of irrigation. For clarity the responses of the *atmyb60-C30 *and *atmyb60-C60 *transgenic lines have been plotted in two different graphs. Each point indicates mean ± SD (n = 20).

We performed an *in-vitro *assay to evaluate the aperture of the stomatal pore in epidermal strips excised from mutant and transgenic lines. In agreement with a previous report [10], light-induced stomatal opening was reduced in the *atmyb60-1 *mutant compared to the wild-type (Figure 4A and 4B). Mutant lines expressing the *VvMYB30 *gene did not display significant differences in the aperture of the stomatal pores compared to *atmyb60-1 *(Figure 4A). Conversely, all the mutant lines transformed with the *VvMYB60 *gene displayed a wild-type response in terms of light-induced stomatal opening, indicating full complementation of the *atmyb60-1 *mutation (Figure 4B).

To substantiate the results obtained *in vitro*, we investigated the effect of both *VvMYB30 *and *VvMYB60 in vivo*, by estimating whole-plant transpiration under stress conditions. Wild-type, *atmyb60-1*, *atmyb60-C30 *and *atmyb60-C60 *plants were grown in soil and pots were covered with tin foil to prevent evaporation, so that water loss occurring through stomatal transpiration could be quantified. Pots were regularly watered for 20 days, and subsequently drought stress was imposed by terminating irrigation. As expected, transpirational water loss, as determined by soil water content measurements, was significantly reduced in *atmyb60-1 *compared to the wild-type (*P < 0.01 *at 2, 4 and 10 days, *P < 0.001 *at 6, 8, 12-18 days) (Figure 4C and 4D). Consistently with results from the *in vitro *assay, mutant lines expressing the *VvMYB30 *gene did not show any difference in term of water loss compared to the *atmyb60-1 *mutant (Figure 4C). Conversely, under the same conditions, the lines expressing the *VvMYB60 *gene displayed a rate of water loss indistinguishable from the one observed in the wild-type, thus demonstrating complete rescue of the stomatal defects of the *atmyb60-1 *mutant (Figure 4D).

## Discussion

### Identification of a Grape ortholog of the AtMYB60 Transcription Factor

The MYB superfamily constitutes the most abundant group of transcription factors found in plants, with at least 198 members in Arabidopsis and 183 in rice [26]. In grape, 108 putative R2R3- MYB family genes were found in the first genome version (8x coverage) [12], whereas more than 125 R2R3 MYB genes can be found using the 12x version (Matus, unpublished results). Plant R2R3-type MYB transcription factors are implicated in several processes related to cell fate, plant development, hormonal responses, pathogen-disease resistance, drought and cold tolerance, light sensing and flavonoid biosynthesis, among many other functions [27]. MYB genes have been intensively investigated in grape, yet most studies have focused on members of the R2R3 clade involved in the regulation of the anthocyanin and pro-anthocyanidin biosynthetic pathway, as the accumulation of these flavonoid compounds in fruit tissues is a key determinant of berry and wine quality [28]. Conversely, MYB genes clustered outside the flavonoid biosynthesis functional group received little attention.

This work shows the identification of the grape *VvMYB60 *gene, as a functional ortholog of the Arabidopsis *AtMYB60 *gene, involved in the regulation of light-induced stomatal aperture [10]. Four lines of evidence support this conclusion: *i*) the aminoacidic sequence of the VvMYB60 and AtMYB60 proteins is highly conserved, *ii*) the *VvMYB60 *and *AtMYB60 *genes show very similar expression profiles, both in terms of tissue- and cell-specificity and response to ABA, *iii*) the *VvMYB60 *and *AtMYB60 *promoters drive expression of reporter genes exclusively in guard cells and share common *cis*-regulatory elements, *iv*) the expression of *VvMYB60 *in the *atmyb60-1 *mutant background completely rescues the loss of the *AtMYB60 *function.

The Arabidopsis and grape MYB60 proteins resulted more similar to each other than to any other MYB in grape or Arabidopsis, even inside subgroup 1, reason why we denoted subgroups 1.1 and 1.2 for further classification. Two main features discriminate between the Arabidopsis and grape MYB60 proteins and other closely related proteins from subgroup 1: a distinctive PHEEG signature in the MYB domain, located in the loop connecting the R2 and R3 repeats, and the lack of the first (CtM1) of three C-terminal motifs present in all the other MYB proteins assigned to subgroup 1 (Figure 1B). Notably, both characteristics are conserved in putative MYB60 orthologs that we identified in other plant genomes, including oilseed rape, tomato, cucumber and poplar (data not shown). Even though a role for the PHEEG and CtM1 motifs has not yet been described, it is intriguing to speculate that the presence of the former and the absence of the latter, might contribute to the specificity of the MYB60 function in guard cells.

### Expression features of *VvMYB60 *in grape organs

It has been previously shown that the Arabidopsis *AtMYB60 *gene is expressed in seedlings, rosette leaves, stems and flowers and its level of expression is rapidly down-regulated by the stress hormone ABA [10]. In addition, publicly available repositories of microarray-based gene profiling experiments indicate that *AtMYB60 *is transiently expressed during seed development, peaking in stage 7 seeds (walking stick embryos) and rapidly declining in mature seeds (The Bio-Array Resource for Plant Functional Genomics, http://bar.utoronto.ca/).

Our survey of *VvMYB30 *and *VvMYB60 *expression in grape tissues revealed that both genes are preferentially expressed in leaves, berry skin and seeds (Figure 2A). Similarly to *AtMYB60*, and opposite to *VvMYB30*, expression of *VvMYB60 *in seeds was down-regulated during seed development (Figure 2B). In berry skin *VvMYB60 *expression was higher before *veraison*, when the grape berry is photosynthetically active and stomata are functional, and was reduced after *veraison*, when stomata evolve into non-functional lenticels [29] (Figure 2C). Interestingly, at this stage, the onset of ripening and the accumulation of sugars are correlated to increasing levels of ABA in the berry [30], suggesting a possible negative effect of the hormone on the expression of *VvMYB60 *in grape tissues. Indeed, treatment of leaves with exogenous ABA resulted in the rapid down-regulation of *VvMYB60 *expression (Figure 2D). In contrast, the hormone did not have any effect on the accumulation of the *VvMYB30 *transcripts (Figure 2E). Additionally, osmotic stresses which trigger ABA-mediated responses, as high concentrations of NaCl, caused the rapid down-regulation of *VvMYB60 *expression in grape tissues (Figure 2F). Interestingly, it has been recently shown that applications of low concentrations of ABA can trigger a transient up-regulation of *MYB60 *expression in Arabidopsis seedlings [31]. This suggests that the pattern of *AtMYB60 *expression in response to osmotic stress might be rather complex and dose-dependent. Even though the detailed analysis of the mechanisms that regulate the expression of the *VvMYB60 *gene extends beyond the scope of this work, it will be intriguing to further investigate the expression profile of *VvMYB60 *in different grape tissues in response to a wider range of ABA concentrations.

### The *VvMYB60 *promoter specifically drives reporter gene expression in Arabidopsis guard cells

Reporter gene analysis and RT-PCR experiments performed on purified Arabidopsis stomata, clearly demonstrated that in green tissues, *AtMYB60 *is exclusively expressed in guard cells [8,24].

We produced Arabidopsis lines harboring the *GUS *marker gene under the control of the *VvMYB30 *and *VvMYB60 *promoters to establish the cellular localization of gene expression. Histochemical analysis of *GUS *expression in several independent lines indicated that the activity of the *VvMYB60 *promoter is restricted to guard cells (Figure 3D, E and 3F). This result is consistent with the expression of the endogenous *VvMYB60 *gene in leaves and berry skin, which both contain stomata, and with the lack of expression in roots (Figure 2A). Reporter gene approaches in Arabidopsis provide efficient and reliable tools to investigate the expression of grape genes and to identify gene regulatory elements [25]. Yet, we did not observe reporter activity in developing seeds of the *pVvMYB30:GUS *and *pVvMYB60:GUS *lines (Additional file 3C). This finding is in contrast with data from qPCR experiments, which showed that both genes are highly expressed in grape seeds (Figure 2A and 2B). This discrepancy could simply be artefactual, because of the heterologous genetic background. However, it is important to note that we did not detect GUS activity in developing seeds of *pAtMYB60:GUS *plants, used as a positive control in this study, despite the high expression of the endogenous Arabidopsis gene in these organs [32]. Different hypotheses can be formulated to explain the lack of activity of the *AtMYB60 *and *VvMYB60 *promoters in seeds. First, *cis*-elements responsible for the seed expression of the endogenous genes could be located outside the regulatory genomic regions considered in this work. However, Cominelli and colleagues reported that the complete 5' and 3' *AtMYB60 *intergenic regions, cloned upstream and downstream of the *GUS *gene, do not drive expression of the reporter in seeds [10]. Alternatively, expression of *AtMYB60 *and *VvMYB60 *in seeds could be mediated by intragenic regulatory elements. *Cis*-acting motifs, located in introns, have been demonstrated to be required to establish the correct expression domain of transcription factors, such as the MADS-box *AGAMOUS *gene [33]. Most interestingly, seed-specific enhancers have been mapped in the intronic regions of seed-expressed genes in different plant species [34]. Finally, the finding that the *AtMYB60 *mRNA is associated with polyribosomes purified from guard cells but not from other plant tissues [35], opens the possibility for a translational level of regulation for MYB60 expression in seeds. Clearly, more work is needed to unravel the nature of the *cis*-regulatory elements that modulate *MYB60 *expression in seeds, together with revealing the function of this gene in these organs. Nevertheless, it is reasonable to conclude that the stomata-specific activity of the *VvMYB60 *promoter in Arabidopsis mirrors the expression of the endogenous gene in grape guard cells.

While the identity of the *cis*-acting elements required for the expression of *AtMYB60 *and *VvMYB60 *in seeds remains elusive, their expression in stomata is most likely regulated, in *cis*, by DOF recognition DNA motifs. We identified a cluster of [A/T]AAAG DOF target sites in close proximity to the *VvMYB60 *translational start codon (Additional file 4). Such a cluster has been described as a guard cell-specific *cis*-regulatory element in different plant species, including Arabidopsis and potato [23,24]. The occurrence of [A/T]AAAG motifs in the guard cell-specific *VvMYB60 *and *VvSIRK *grape promoters lends further support to the conservation of the *cis*- and, possibly, *trans-*mechanisms that direct expression in guard cells in distantly related plant species. Interestingly, strong conservation across a wide range of flowering plant species has also been reported for other cell-specific *cis*-motifs, such as the root hair-specific *cis*-elements (RHEs) [36].

### *VvMYB60 *is a functional ortholog of *AtMYB60*

The ability of VvMYB60 to fully complement, both *in vitro *and *in vivo*, the stomatal defects exhibited by the *atmy60-1 *mutant unequivocally demonstrates that VvMYB60 is a true ortholog of the Arabidopsis AtMYB60 transcription factor. Importantly, the *VvMYB30 *gene product, which shares 47% identity to *VvMYB60*, did not complement the *atmyb60-1 *mutation. This result is in agreement with functional studies which indicate that, despite the high degree of homology between the AtMYB30 and AtMYB60 aminoacidic sequences, these two proteins play two distinct functional roles. In Arabidopsis, AtMYB30 mediates brassinosteroid-induced gene expression [15] and pathogen-induced hypersensitive response [16], whereas AtMYB60 positively regulates light-induced stomatal opening and modulates water loss under drought [10].

As a whole, our findings indicate a role for VvMYB60 in the regulation of guard cell activity and transpiration rate in grapevine. Stomatal conductance is a key trait in grapevine, as it directly determines the isohydric/anisohydric behavior displayed by different genotypes. These differences are due to stomatal control over evaporative demand rather than stomatal density in vegetative tissues [37]. Cultivar-specific differences have also been described for the effects of water deficit on ABA metabolism and signaling [38]. Anisohydric cultivars such as Pinot Noir possess insufficient stomatal regulation and show high transpiration rates and stomatal conductance, whereas isohydric cultivars as Shiraz, display much lower values [39]. In this perspective, it will be interesting to survey variations in naturally occurring *VvMYB60 *alleles and to establish their contribution to differences in stomatal activity in different Vitis species and cultivars.

## Conclusions

*VvMYB60 *could represent a valuable target for downstream biotechnological applications. Although grapevine is a highly productive water stress-adapted plant, the availability of molecular targets for engineering or breeding of new cultivars with enhanced stomatal responses represents an attractive approach to increase water use efficiency and, possibly, to reduce pathogen penetration through the stomatal pore [40,41].

## Methods

### Phylogeny reconstruction and bootstrap analysis

Alignments were performed using the BLOSUM matrix (Gap opening and extension penalties of 10 and 5, respectively) of the ClustalW algorithm-based AlignX^® ^module from Mega4 Software [42]. A rooted tree was constructed using the Neighbour Joining Method (NJ) in MEGA4 and confirmed with MEGA3. Tree nodes were evaluated by bootstrap analysis for 100 replicates (pairwise deletion, uniform rates and Poisson correction options). Publicly available sequences were collected from Genbank via NCBI (http://www.ncbi.nlm.nih.gov/). The corresponding cDNAs of the complete coding sequences of *VvMYB30 *and *VvMYB60 *were amplified from PN40024 genomic DNA and leaf cDNA, respectively, using the following primer combinations: VvMYB30F1-VvMYB30R3 and Vv60L2F4-Vv60L2R4 (see below for primer sequences).

### Field sampling of grape organs and nucleic acid extraction

Grapevine organs (*Vitis vinifera *L. cv. Cabernet Sauvignon) were collected from a commercial vineyard in the Maipo Valley, Chile, and frozen in liquid nitrogen for RNA extraction. For grape berry skin and seed sampling, a total of nine grape clusters were collected from three plants every two weeks during fruit development, beginning two weeks after fruit set and ending at eight weeks after *veraison*. Total RNA was isolated from all grapevine tissues as described [43]. For cDNA synthesis, one μg of total RNA was reverse transcribed with random hexamer primers using StrataScript^® ^reverse transcriptase (Stratagene) according to the manufacturer's instructions.

### ABA and salt treatment experiments

For ABA treatments, young leaves of *Vitis vinifera *cv. Cabernet Sauvignon were cut from two month old plantlets cultivated *in vitro *and placed in petri dishes supplemented with 50 μM, 100 μM ABA (+/- *cis*, *trans *ABA; SIGMA), dissolved in 100% ethanol, or with an equal amount of 100% ethanol (mock solution). Samples were maintained in a growth chamber at 20°C in the light (120 μmol m^-2 ^sec^-1 ^of measured light irradiance), and every two hours three leaves from each treatment were collected for RNA extraction.

For salt treatments, nodal explants of *Vitis vinifera *cv. Cabernet Sauvignon were placed vertically on sterile MS medium supplemented with 3 mM NaCl (standard), 100 mM NaCl or 200 mM NaCl and left for a month in a growth chamber (20°C; 16 h photoperiod). At the end of the experiment, complete plantlets were collected for RNA extraction.

### Quantitative comparison of gene expression in grape tissues

Relative transcript quantification of isolated genes was achieved by real time RT-PCR, using the Brilliant^® ^SYBR^® ^Green QPCR Master Reagent Kit (Stratagene) and the Mx3000P detection system (Stratagene) as described in the manufacturer's manual. Primers qPCR_VvMYB30fw (5'-CTCAAGTCCCTCTCACAATG-3') and qPCR_VvMYB30rev (5'-TGTCAATTAGGTCTTCTTGTTC-3'), qPCR_VvMYB60fw (5'-TTGAGTACGAAAACCTGAATGAT-3') and qPCR_VvMYB60rev (5'-GGAGGGTTGTGCTTCTTCTGAT-3') were used for amplification and qPCR quantification of *VvMYB30 *(81 bp) and *VvMYB60 *(121 bp), respectively. Amplification of the *UBIQUITIN *(99 bp) or *GLYCERALDEHYDE 3-PHOSPHATE DEHYDROGENASE *(*G3PDH*) genes was used for normalization [44], depending on their expression variations for each experimental condition. PCR conditions, standard quantification curves for each gene and relative gene expression calculations were conducted according to Matus *et al*. [45].

### Plasmid constructs, generation and analysis of Arabidopsis transgenic lines

To generate the *pVvMYB30:GUS *construct, a region of 2,173 bp upstream of the translational start codon was amplified from grape genomic DNA (Pinot noir, PN40024), using primers VvMYB30F3 (5'-AAGCTTCTGACGCAGTTTTCAACCATC-3'), containing a HindIII site, and VvMYB30R4 (5'-TCTAGAGGTGGCCTCCCCTTGGCT-3'), containing an XbaI site. The HindIII-XbaI fragment was cloned upstream of the *uidA *coding sequence in the pBI101.3 binary vector (Stratagene). Similarly, the 2,239 bp putative *pVvMYB60:GUS *promoter was amplified with primers Vv60F3 (5'-AAGCTTATGAGAGGTCGTATAAGTA-3'), containing a HindIII site, and Vv60R3 (5'-TCTAGAGGCCTTCCTATGGCTT-3'), containing an XbaI site, and the PCR fragment was cloned in the pBI101.3 vector. The *VvMYB30 *full length cDNA, was obtained by amplification of PN40024 leaf cDNA with the primers VvMYB30F1 (5'-GGATCCATGGGGAGGCCACCTTG-3'), containing a BamHI site and VvMYB30R3 (5'-GATATCTAGAAGAGCTGAGCAGTGTCCT-3'), containing an EcoRV site. The full length *VvMYB60 *cDNA was amplified with the primer Vv60L2F4 (5'-GGATCCATGGGAAGGCCTCCTTGCTGTG-3'), containing a BamHI site and the primer Vv60L2R4 (5'-GAGCTCTCAGAATATTGGAGAGAGTTGATCC-3'), containing a SacI site. Amplified cDNAs were sequenced and then cloned in a modified version of the pPZP221 binary vector, containing the 1.3 kb *AtMYB60 *promoter and the *nos *terminator (Galbiati, unpublished). All constructs were introduced in *Arabidopsis *(Col-0) by *Agrobacterium*-mediated transformation as described [22]. Transformed lines were selected on antibiotic containing media, and the presence of the transgene was confirmed by PCR. For analysis of transgene expression, total RNA was isolated with the RNeasy Mini Kit (Qiagen) and reverse-transcribed with the RT Superscript II kit (Invitrogen). Semi-quantitative RT-PCRs were performed for 25 cycles with primer pairs: Vv30F2 (5'-GGATCCATGGGAAGGCCTCCTTGCT-3') and Vv30R2 (5'-AAGTCTGACAGTGATGAGAGGAGC-3'); Vv60F2 (5'-CTCCTTGCTGTGATAAAGTTGGTAT-3') and Vv60R2 (5'-ATTCAGGTTTTCGTACTCAAGAATG-3'). The control *AtACTIN2 *gene (At3g18780) [46] was amplified using primers AtACT2F (5'-GTGTTGGACTCTGGAGATGGTGTG-3') and AtACT2R (5'-GCCAAAGCAGTGATCTCTTTGCTC-3'). Homozygous T3 lines were selected for GUS staining and functional complementation analyses.

### Arabidopsis growth, ABA treatment and analysis of GUS expression

Seeds were surface-sterilized overnight in a sealed chamber in the presence of 100 ml of commercial bleach and 3 ml of 37% HCl, and germinated in Petri dishes containing Murashige and Skoog medium, 1% w/v sucrose and 0.8% w/v agar. Plants were grown under long-day conditions (16 h light/8 h dark, at 100 μmol m^-2 ^sec^-1^) at 22°C in a controlled growth chamber. For ABA treatments, plants were transferred to liquid MS medium with 3% w/v sucrose and 0.5 g/l MES, supplemented with 100 μM ABA (+/- *cis*, *trans *ABA; SIGMA), dissolved in 100% ethanol, or with an equal amount of 100% ethanol (mock solution). For detection of GUS activity, tissues were incubated for 6 hr, at 37°C, in 0.5 mg/ml X-glucuronic acid, 0.1% Triton X-100, and 0.5 mM ferrocyanidine in 100 mM phosphate buffer (pH 7). Tissues were cleared with 70% ethanol and examined using a Leica M205 FA stereoscope or a Leica DM2500 optical microscope. qPCR analysis of GUS expression was performed as described for the grape samples, using primers qPCR_GUSF1 (5'-TACGGCAAAGTGTGGGTCAATAATCA-3') and qPCR_GUSR1 (5'-CAGGTGTTCGGCGTGGTGTAGAG-3'). GUS expression was normalized using the control *AtACTIN2 *gene (At3g18780) [46], amplified with primers qPCR_AtACT2fw (5'-TGCTTCTCCATTTGTTTGTTTC-3') and qPCR_AtACT2rev (5'-GGCATCAATTCGATCACTCA-3').

### Stomatal aperture and water loss measurements

Stomatal assays were performed on abaxial epidermis strips, incubated in 30 mM KCl, 10 mM MES-KOH, pH 6.5, at 22°C, and exposed to light (300 μmol m^-2 ^sec^-1^) for 4 h. Measurements of stomatal aperture were performed using a Leica DM2500 optical microscope and the LAS Image Analysis software. For drought experiments, seeds were germinated in individual pots each containing the same amount of pre-wetted soil. Plants were regularly irrigated for 20 days. Before watering was terminated, pots were covered with tin foil to minimize evaporation from soil. Pots were weighed every other day at the same time for 18 days. At the end of the treatment, pots were dried for three days at 65°C to determine the dry weight. Water content was estimated as [(Wt_n_-DW)/(Wt_0_-DW)] × 100, where Wt_n _= total weight of the pot at day n; DW = dry weight of the pot and Wt_0 _= total weight of the pot at day 0.

## Authors' contributions

MG and JTM contributed to the conception of the study, drafted the manuscript, carried out the grape genome search and cloning of MYB60 like genes and their promoter sequences. MG, PF, FR, LC and EC, produced and analysed the Arabidopsis transgenic lines described in the work. JTM carried out the phylogenetic study and, together with PC and CM, tested *VvMYB60 *and *VvMYB30 *expression in *Vitis vinifera *organs and experimental conditions. CT and PAJ were involved in revising the manuscript critically for important intellectual content and gave final approval of the version to be published. All authors read and approved the final manuscript.

## Supplementary Material

Additional file 1 **Deduced gene structure of *AtMYB60*, *VvMYB30 *and *VvMYB60***. Boxes represent exons, while black lines represent introns. The location of the ATG start codon is indicated (black arrow). Gene organization and size of exons and introns were deduced by comparing the sequence of amplified genomic and cDNA fragments. Yellow and green boxes represent exon sequences coding for the R2 and R3 repeat, respectively. Click here for file

Additional file 2 **Phenotypic changes in grapevine plantlets grown in the presence of growing NaCl concentration**. Pictures were taken one month after the beginning of the treatment. Click here for file

Additional file 3 **Activity of the grape *VvMYB360 *and *VvMYB60 *promoters in flowers and siliques from Arabidopsis lines carrying promoter:GUS fusions**. (**A**) GUS expression in *pVvMYB30:GUS *flowers was localized in carpels and stigmatic tissues (arrow). (**B**) Most *pVvMYB60:GUS *flowers did not show GUS activity, with the exception of two independent lines which disclosed staining in the distal part of the anther filament (arrow). (**C**) *pVvMYB60:GUS *siliques did not show GUS expression in developing seeds (Bars = 1 mm). Click here for file

Additional file 4 **Occurrence of [A/T]AAAG motifs in the 300 bp regulatory region located upstream of the translational start codon of the *AtMYB60*, *VvMYB30*, *VvMYB60 *and *VvSIRK *genes**. [A/T]AAAG nucleotides on the + strand are highlighted in yellow, whereas [A/T]AAAG nucleotides on the - strand are highlighted in pale blue. The predicted TATA box is in italic and highlighted in green, the ATG codon is highlighted in dark blue. Sequences encompassing clusters of [A/T]AAAG motifs (see text for definition) are in bold and underlined. Click here for file

Additional file 5 **Generation and selection of the transgenic lines used for the complementation of the *atmyb60-1 *Arabidopsis mutant (*atmyb60-C60 *and *atmyb60-C30*)**. (**A**) and (**B**), schematic representation of the constructs used in the complementation test (not to scale). (**C**) and (**D**), RT-PCR analysis of transgene expression (*VvMYB60 *and *VvMYB30*) in three independent homozygous T3 transformed *atmyb60-1 *lines. (), lane 1 = *atmyb60-C60-1*; lane 2 = *atmyb60-C60-2*; lane 3 = *atmyb60-C60-3*; lane 4 = *atmyb60-1*; lane 5 = dH_2_O. (**D**), lane 1 = dH_2_O; lane 2 = *atmyb60-C30-1*; lane 3 = *atmyb60-C30-2*; lane 4 = *atmyb60-C30-3*; lane 5 = *atmyb60-1*. The Arabidopsis *AtACTIN2 *gene (At3g18780) was used as a control. Click here for file
