# Supplementary figures and images for: The grapevine guard cell-related VvMYB60 transcription factor is involved in the regulation of stomatal activity and is differentially expressed in response to ABA and osmotic stress
Source: BMC Plant Biol. 2011 Oct 21;11:142. doi: 10.1186/1471-2229-11-142 (PMC3206852; doi:10.1186/1471-2229-11-142)

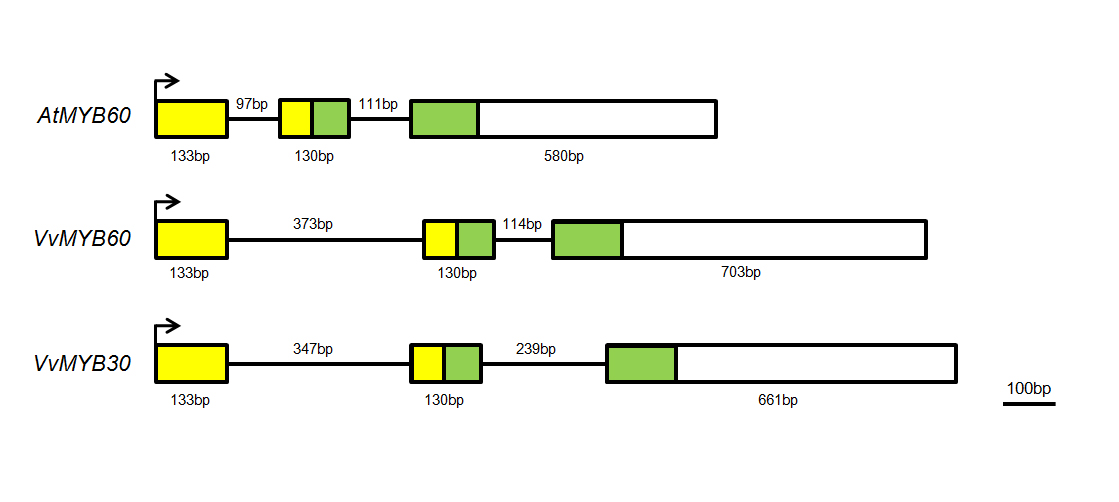

Supplement: Additional file 1 — Deduced gene structure of AtMYB60, VvMYB30 and VvMYB60. Boxes represent exons, while black lines represent introns. The location of the ATG start codon is indicated (black arrow). Gene organization and size of exons and introns were deduced by comparing the sequence of amplified genomic and cDNA fragments. Yellow and green boxes represent exon sequences coding for the R2 and R3 repeat, respectively. [file 1471-2229-11-142-S1.JPEG]

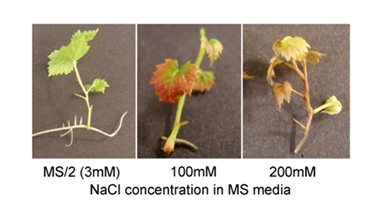

Supplement: Additional file 2 — Phenotypic changes in grapevine plantlets grown in the presence of growing NaCl concentration. Pictures were taken one month after the beginning of the treatment. [file 1471-2229-11-142-S2.JPEG]

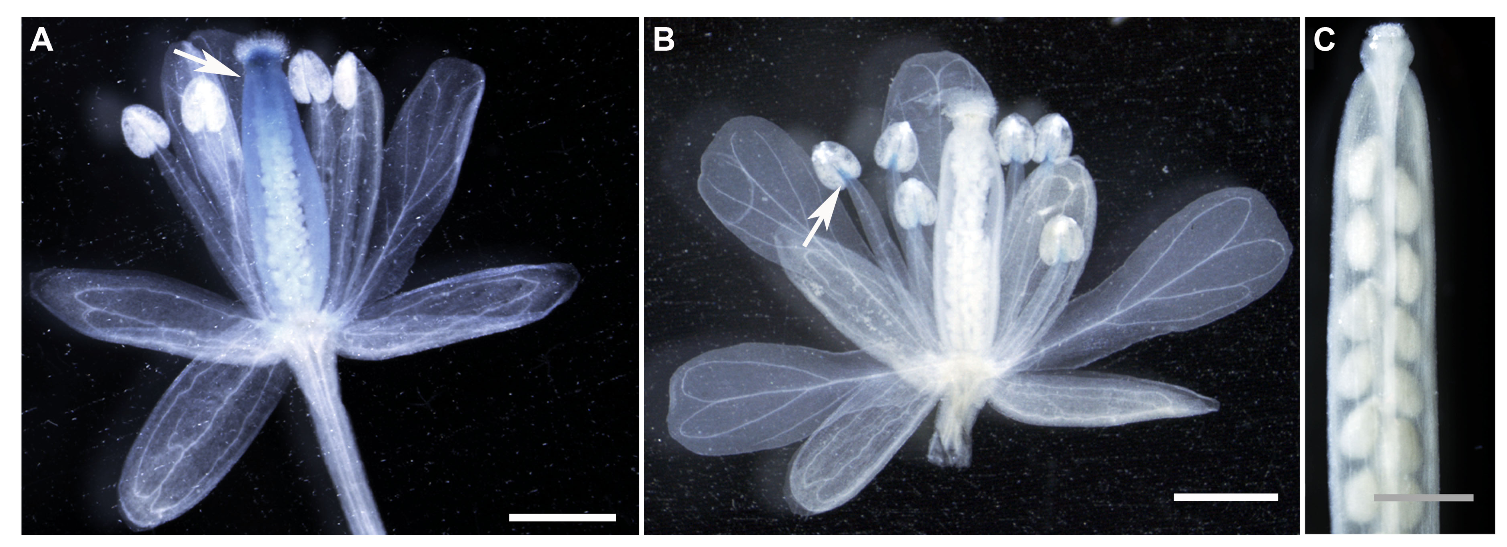

Supplement: Additional file 3 — Activity of the grape VvMYB360 and VvMYB60 promoters in flowers and siliques from Arabidopsis lines carrying promoter:GUS fusions. (A) GUS expression in pVvMYB30:GUS flowers was localized in carpels and stigmatic tissues (arrow). (B) Most pVvMYB60:GUS flowers did not show GUS activity, with the exception of two independent lines which disclosed staining in the distal part of the anther filament (arrow). (C) pVvMYB60:GUS siliques did not show GUS expression in developing seeds (Bars = 1 mm). [file 1471-2229-11-142-S3.TIFF]

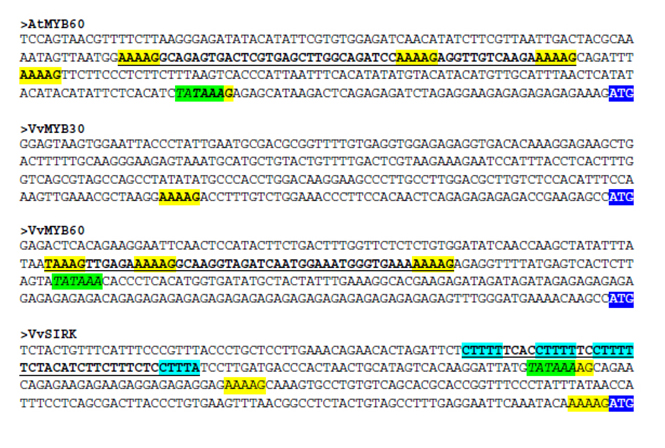

Supplement: Additional file 4 — Occurrence of [A/T]AAAG motifs in the 300 bp regulatory region located upstream of the translational start codon of the AtMYB60, VvMYB30, VvMYB60 and VvSIRK genes. [A/T]AAAG nucleotides on the + strand are highlighted in yellow, whereas [A/T]AAAG nucleotides on the - strand are highlighted in pale blue. The predicted TATA box is in italic and highlighted in green, the ATG codon is highlighted in dark blue. Sequences encompassing clusters of [A/T]AAAG motifs (see text for definition) are in bold and underlined. [file 1471-2229-11-142-S4.JPEG]

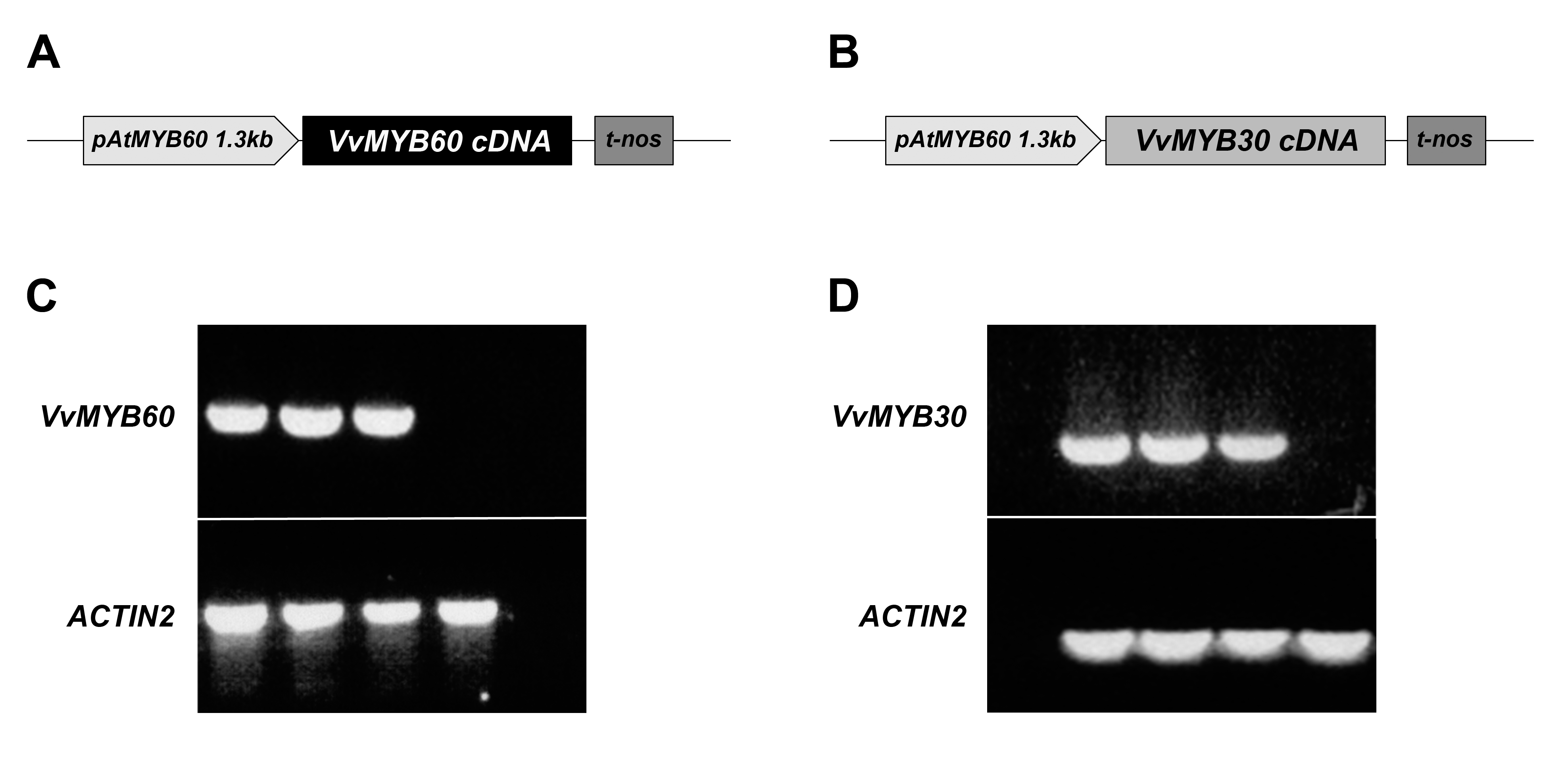

Supplement: Additional file 5 — Generation and selection of the transgenic lines used for the complementation of the atmyb60-1 Arabidopsis mutant (atmyb60-C60 and atmyb60-C30). (A) and (B), schematic representation of the constructs used in the complementation test (not to scale). (C) and (D), RT-PCR analysis of transgene expression (VvMYB60 and VvMYB30) in three independent homozygous T3 transformed atmyb60-1 lines. (), lane 1 = atmyb60-C60-1; lane 2 = atmyb60-C60-2; lane 3 = atmyb60-C60-3; lane 4 = atmyb60-1; lane 5 = dH2O. (D), lane 1 = dH2O; lane 2 = atmyb60-C30-1; lane 3 = atmyb60-C30-2; lane 4 = atmyb60-C30-3; lane 5 = atmyb60-1. The Arabidopsis AtACTIN2 gene (At3g18780) was used as a control. [file 1471-2229-11-142-S5.TIFF]
